# Liquor Ipecacuanhæ Et Morphinæ—Dover’s Solution

**Published:** 1885-07-20

**Authors:** Edwin H. Hess


### LIQUOR IPECACUANHÆ ET MORPHINÆ.—DOVER'S SOLUTION.

By Edwin H. Hess, Ph. G.

From an Inaugural Essay.

### Preparation.

Take of acetate of morphine one drachm; diluted acetic acid, one fluidounce; diluted alcohol, seven fluidounces; wine of ipecac, two fluidounces. Dissolve the acetate of morphine in the acid, add the diluted alcohol and wine of ipecac, and mix the whole thoroughly. Set aside for twenty-four hours, then filter through paper.

This preparation is not generally known, although used quite extensively where it has been introduced. It originated with Dr. J. D. Coleman of Juliustown, near Mount Holly, Burlington county,

N. J., and afterward of Trenton, N. J., now deceased. The preparation is used in only a few other places, as far as I can ascertain; but, judging from its popularity in those places, it certainly deserves a much wider scope for usefulness. In Trenton, where I became acquainted with it, it may be gotten from any pharmacist (and is always kept in stock), is prescribed by almost all the physicians, and has a local reputation among the people as a remedy for all ills common to mankind, probably as great as paregoric.

It is transparent, of a yellowish or amber color, due to the coloring matter of the ipecac and the wine. At first the odor is decidedly vinous, but at the same time acetous. It changes rapidly, however, becoming more agreeable, and then somewhat resembles the odor of whisky, and doubtless contains some of the same or allied ethers. It has very little taste other than the persistent bitter of the morphine. Three ounces by measure should weigh exactly 2¾ ounces troy, making its specific gravity .9654. Its color is very little affected by sunlight and refracted light. It has an acid reaction with test paper. It enters into the composition of a preparation known as

RED DROPS,

Viz.: Tinctura catechu composita, ℥v; spiritus camphoræ, ℥j; liquor Doveri, ℥ij. This is quite an efficient remedy for diarrhœa, dysentery, cholera morbus, and all summer complaints in general. Locally, it has acquired no mean reputation among the physicians and others, and it is thought by some to be equally as good, if not superior, to the renowned Asiatic cholera mixture, Squibb's compound solution of opium, and other preparations of wide-spread reputation.

In addition to the Dover's powder, a new preparation, known as tincture of ipecac and opium was made official in 1880. The solution of acetate of morphia (B. P.) is somewhat similar in its anodyne properties to the Dover's solution, and contains the acetate of morphine in acid solution of the strength of ½ gr. to the drachm. Dover's syrup is a preparation manufactured in Philadelphia, containing syrup as a menstruum, which makes it quite palatable. Syrup, however, is apt to prove disadvantageous on account of its nauseating effects. Nearly all the preparations of this type have this effect, from the fact that they are compounds of opium, and not morphine.

The medical properties accorded to the "solution" are, diuretic, diaphoretic, analgesic and sedative. It is quite efficient in the treatment of coughs, colds, etc., and in the first stages of acute inflammations attending throat and lung troubles. It is also very useful in the treatment of rheumatism, neuralgia, etc.

A fluidounce evaporated to pilular consistence should not weigh more than 7½ grs. (.486 gms.). In attempting to scale the residue by the usual method I was not successful on account of its extreme deliquescence. The resulting mass, however, remains stable for several months, and may be conveniently rolled into pills. From several experiments, I found it to be quite as effective in this form as in solution, and being quite heavy (dose, ½ grain), the pellets are very easy to take. The solution, on standing, without having been filtered, deposited a dark-colored sediment, light in weight. On testing this for morphine none was found. A full grown cat was given different doses (of both liquid and solid form of the preparations), ranging from $\frac{1}{8}$ gr. to two grs., with the effect of producing sleep. Larger doses produced emesis, with general exhaustion, but no alarming symptoms. From these experiments, I concluded that it is almost a harmless preparation, as an overdose is almost certain to produce emesis.—*Amer. Jour. Pharm.*

